# Selective Reporting of Outcomes in Tinnitus Trials: Comparison of Trial Registries With Corresponding Publications

**DOI:** 10.3389/fneur.2021.669501

**Published:** 2021-06-10

**Authors:** Isabeau van Beurden, Megan J. van de Beek, Jan A. A. van Heteren, Adriana L. Smit, Inge Stegeman

**Affiliations:** ^1^Department of Otorhinolaryngology and Head and Neck Surgery, University Medical Center Utrecht, Utrecht, Netherlands; ^2^Department of Clinical and Experimental Neuroscience, University Medical Center Utrecht Brain Center, Utrecht University, Utrecht, Netherlands

**Keywords:** tinnitus, reporting academic misconduct, outcomes, bias, trials

## Abstract

**Objectives:** We aimed to study the prevalence of selective reporting of primary and secondary outcomes in tinnitus trials and to examine if selective reporting of outcome measures is influenced by the nature and direction of its results.

**Background:** Selective reporting of outcome measures has been reported in several biomedical fields and can influence the clinical usefulness and implementation of outcomes of clinical trials. It is reported as one of the obstacles in finding an effective intervention for tinnitus.

**Methods:** ClinicalTrials.gov (CT.gov) was used to identify all registered interventional tinnitus trials up to December 2015. A standardized search was used to find corresponding publications up to March 2018. The prespecified outcomes in CT.gov were compared with the outcomes reported in corresponding publication(s). The effects of the (lack of) statistical significance of trial results and the effects of funding source on record adherence were evaluated. Changes in registration elements were assessed with the Archive site of CT.gov.

**Results:** We found corresponding publications for 60 (64.5%) of 93 eligible tinnitus trials registered in CT.gov. Of all the publications, five (7.5%) fully reported outcome measures entirely in line with the prespecified outcome measures. Discrepancies between the prespecified and reported outcomes were found in a total of 51 (76.1%) of the studies for primary outcomes, whereas 62 (92.5%) of the studies had discrepancies in secondary outcomes. In secondary outcomes, statistical significance of trial results influenced CT.gov record adherence. In addition, there was a statistically significant difference in the rate of discrepancy in industry-funded [*n* = 98 (87.5%) discrepant outcomes] and non-industry funded trials [*n* = 172 (74.5%) discrepant outcomes] (*p* = 0.01). Finally, 15 (25.9%) trialists made modifications in registered outcome measures during or after the trial period.

**Conclusion:** Tinnitus trials suffer from substantial outcome reporting bias. Awareness of its presence must be raised to limit the obstacles of finding an effective intervention for tinnitus.

## Introduction

Tinnitus is characterized by the perception of sound in the absence of an external stimulus (1). It is a common condition, and the burden of disease differs between individual patients. It can have severe impact on quality of life by impairing thought processing, hearing, sleep, emotions, and concentration (2). Despite the efforts of researchers and clinicians, till date, there is no curative treatment. The quality of research and reporting of robust outcome measures in tinnitus studies is recognized as one of the obstacles in finding an effective intervention (3).

In an era of evidence-based medicine (EBM), information in scientific publications must be transparent, openly accessible, complete, and readable, in order for clinicians to draw conclusions (4). Therefore, the expanding number of reports about inaccessible research, research waste, and the incorrect use of research methods and results are worrisome (5). One important part of research waste is reporting bias, which is defined as the selective reporting of prespecified outcome measures, depending on the nature and direction of the analyzed results (6). Reporting bias might be responsible for overestimates of beneficial effects and suppression of harmful effects of treatment (7, 8). It introduces flaws into treatment recommendation and guidelines when results of biased reports are included in meta-analysis (8). In an era of evidence-based medicines, trials and guidelines are important in clinical decision making. Therefore, both clinicians and researchers need to be aware of the influence of reporting bias on trial outcomes.

Trial registration was introduced to improve quality and transparency in methods and results. In July 2005, the International Committee of Medical Journal Editors (ICMJE) stipulated that all investigators must register clinical trials in a qualifying public database before patient enrolment as a condition for publication [i.e., prospective trial registration; (9)]. As part of registering a clinical trial, it is generally required to complete the 20-item minimum dataset of the World Health Organization (WHO), including specifying primary and secondary outcome measures (9). Trial registries also provide a history of changes made to the record after the date it was first submitted (10). While many countries have a specific country trial register, ClinicalTrials.gov (CT.gov) is the largest publicly accessible international trial registry. The obligation to register a trial is part of the transition from “science for ourselves” to open science. Not only trial registration but also the publication of full protocols, open access publications, and willingness to share methodology and data, are part of this science movement.

Selective reporting of outcomes appears as discrepancies between prespecified outcomes reported in the trial registry and those reported in corresponding publications. Prespecified outcomes may be downgraded from primary to secondary outcomes (or vice versa), and outcomes may be omitted from, or newly added to, the corresponding publication based on the nature and direction of the results [e.g., unexpected negative results or (lack of) statistical significance]. Previous studies in different clinical areas have shown that 5.9–46.6% of the published trials had discrepancies in primary outcomes, and 40.0–90.0% of the published trials had discrepancies in secondary outcomes (8, 11–17). Moreover, discrepancies between prespecified and published outcome measures are more often favored by statistically significant results than statistically non-significant results (18).

In a previous study, we investigated the prevalence of publication bias in clinical trials in the research field of otology (19). For only 46.3% of the completed trials, a corresponding publication was found. In the present study, our primary aim was to determine the prevalence of discrepancies in outcome measures in tinnitus studies by comparing preregistered outcomes in CT.gov and reported outcomes in corresponding publications. Second, we aim to determine whether selective reporting of outcomes is favored by (the lack of) statistical significance of trial results or funding source, and to evaluate the history of change in outcome measures in the trial register.

## Methods

### Search Strategy for Eligible Trials in ClinicalTrials.gov

The trial registry ClinicalTrials.gov was searched by one investigator (JH) on January 10, 2017, to identify all registered tinnitus trials. The topic “Ear, Nose, and Throat Diseases” was selected, and all available information was obtained and exported into a spreadsheet. Interventional trials primarily discussing the subject tinnitus in title or study description were included. Observational studies and duplicate-labeled trials [i.e., the same National Clinical Trial (NCT) number registered within different topics, e.g., tinnitus, deafness, vestibular disease] were excluded. Trials completed after December 31, 2015, were omitted in order to provide at least a 2-year period for the investigators to submit an article for publication, peer review, and editorial processes, since the median time to publication in otology trials is 24 months (19). Accordingly, trials that did not disclose a completion date but reported a start date after December 31, 2015 were excluded (Supplementary Dataset 1).

### Search Strategy for Corresponding Publications and Selection Criteria

Two independent investigators (IB and MB) inquired corresponding publications by using a standardized search strategy. Any disagreement about inclusion was resolved by a third investigator (IS). Publications, defined as a complete manuscript in a peer-reviewed journal, were included up to March 2018. The “More information” field on CT.gov was examined for corresponding publications, considering this field is used to acknowledge citations of relevant research or trial results. PubMed and Embase were used to search the Medical Literature Analysis and Retrieval System Online (MEDLINE) for publications, by using the NCT number and/or keywords [e.g., principal investigator(s), trial title, intervention, outcomes, study design, institution, etc.].

Publications, including reports of pilot studies and preliminary results, were selected when matching with the corresponding trial, based on the title, trial description, primary and secondary outcome measures, start and end dates, the number of participants enrolled, country of origin, and author(s). When multiple publications were retrieved for the same trial, all publications were included in the assessment. Letters to the editor, study protocols, abstracts, and studies that reported the NCT number but did not report any outcomes of the trial were excluded. If no publication was found by our extensive search, we considered it as “unpublished.”

### Extraction of Data Elements From ClinicalTrials.gov and Corresponding Publications

All trial data elements were exported from ClinicalTrials.gov, including study design, recruitment status, study phase, funding, type of allocation, patient enrollment, age, trial registration date, “Study start date,” and “Study completion date.” In CT.gov, the “Study start date” is defined as the date at which the first participant is enrolled in a clinical study. “Study completion date” is defined as the date on which the last participant was examined or received an intervention to collect data for the primary and secondary outcome measures. The most recent primary and secondary outcome measures listed in ClincalTrials.gov were obtained on March 18, 2017. The study topic was retrieved manually from title and study description in the trial registry and cross-checked for inconsistency by two independent investigators (IB and MB). Results reported in CT.gov were not taken into consideration since these can be changed over time and are not necessarily the final results. In addition, the statistical significance of the results is not fully reported.

The following data were extracted from corresponding publications: author's e-mail address, reporting of NCT number, publication date (the electronic publication date or when this date was not provided, and the publication date in print), all outcome measures, and the statistical significance of the results. Primary outcomes in either ClinicalTrials.gov records or publications were defined as those that were explicitly reported as “primary outcome,” “main outcome,” or “key outcome.” Accordingly, secondary outcomes were those that were reported as “secondary outcome” or “other outcome.” If the outcome measure was not explicitly identified using these terms, these outcome measures were coded as “undefined.” The results of the outcomes were considered as statistically significant if they were reported as such by the authors [i.e., results reported as “significant,” a *p* < 0.05, or the 95% confidence interval (95% CI) around the observed effect size excluded the no-effect value]. Statistical significance of outcomes was considered as “unclear” when no *p*-value or 95% CI was reported, or when the significance of the results remained undescribed. Both between- and within-group comparisons were taken into consideration when assessing the statistical significance of the reported outcome.

### Assessment of Selective Reporting of Outcomes

Two investigators (IB and MB) independently compared each prespecified registered outcome measure to the reported outcome measures in the publication for consistency. Disagreements were resolved by consensus after reviewing the articles a second time. Unresolved disagreement was reviewed by a third investigator (IS).

Trials reporting no outcome measure in CT.gov could not be used for the assessment of selective outcome reporting. When multiple publications for the same trial were retrieved, each publication was compared separately with the trial protocol, and each publication was considered as an unique trial. Only if multiple publications reported different outcome measures of the same trial were the outcome measures combined, and the publications were considered as one. It is noted that changes can be made in reporting of outcome measures between trial protocol and publication. Consistent with the CONSORT guidelines, when a reason for the discrepancy in outcome measure was described in the publication, it was not disclosed as a discrepancy (20).

The discrepancies between prespecified and reported outcomes were categorized in:

- no discrepancy between prespecified and published outcome measures;
- prespecified outcomes that were unpublished;
- prespecified outcomes that remained undefined in the publication (i.e., neither reported as primary, nor as secondary outcome measure);
- prespecified outcomes that changed from primary to secondary or vice versa in the publication;
- newly published outcomes that were not prespecified on CT.gov, and
- altering the timing of assessment between prespecified and published outcomes.

Although registration quality was not the focus of this study, registered trials lacking a fully reported outcome measure or lacking timing of assessment in CT.gov were reported.

To examine reasons for discrepancy in outcome measures, the authors of the publications were contacted via e-mail using a standardized email template inquiring about a justification for their decision making. If a reply was not received within 2 weeks, authors were contacted a second time. Corresponding publications provided by the authors that were not found by our extensive search were included afterward.

Because trial registries allow investigators to update outcome measures at any point, www.clinicaltrials.gov/archive was used to evaluate the history of changes in outcome measures after trial registration, since this could give insight in selective outcome reporting. Deletion, addition, upgrading, or downgrading of outcome measures were extracted between the first and last updated versions in the database.

### Data Analysis

All trial data elements were categorical, except for two continuous variables (patient enrollment and trial duration). ClinicalTrials.gov categorizes funding as follows: the federal government of the United States (U.S. federal), industry, NIH, and other (including universities, non-profit organizations, and hospitals). In this study, funding was categorized for analysis in “industry” in case any of the listed funding sources included industry, or “non-industry” (including U.S. federal, NIH, and other organizations). The trial registration date, “Study start date” and “Study completion date” were compared to assess whether trials were registered prospectively, during patient enrollment, or retrospectively (9). Accordingly, trial duration was calculated in number of months from “Study start date” to “Study completion date” and time to publication in number of months from “Study completion date” to date of publication. Last, the study topic was categorized in six groups; “Psychology,” “Sound therapy,” “Pharmacology,” “Transcranial Magnetic Stimulation (TMS),” “Other” (e.g., laser therapy, myofascial trigger point release, neurofeedback, etc.) and “Combined” when a combination of different topics was investigated. When any of the data elements were not reported in ClinicalTrials.gov, it was classified as “missing.”

The following descriptive analyses were computed to assess study characteristics and discrepancy rates of primary and secondary outcome measures: number (percent) for discrete variables and median and interquartile range (IQR) for continuous variables. To examine whether the statistical significance of results influences authors to alter prespecified outcome measures, a Chi-square test was used, comparing the discrepancy rate in “significant,” “non-significant,” or “unclear” results (either within- or between-group comparisons). The Chi-square test was also used to analyze record adherence in industry- and non-industry funded trials. To further extricate possible reasons for discrepancy, Pearson correlation was used to assess the correlation between the number of prespecified outcomes and the number of non-reported outcomes in the publication. Differences were considered to be statistically significant if the *p* < 0.05. IBM SPSS statistics for Windows, version 25.0 (IBM Corp., Armonk, NY, USA) was used for analysis.

## Results

### Trial Characteristics

**Figure 1:**
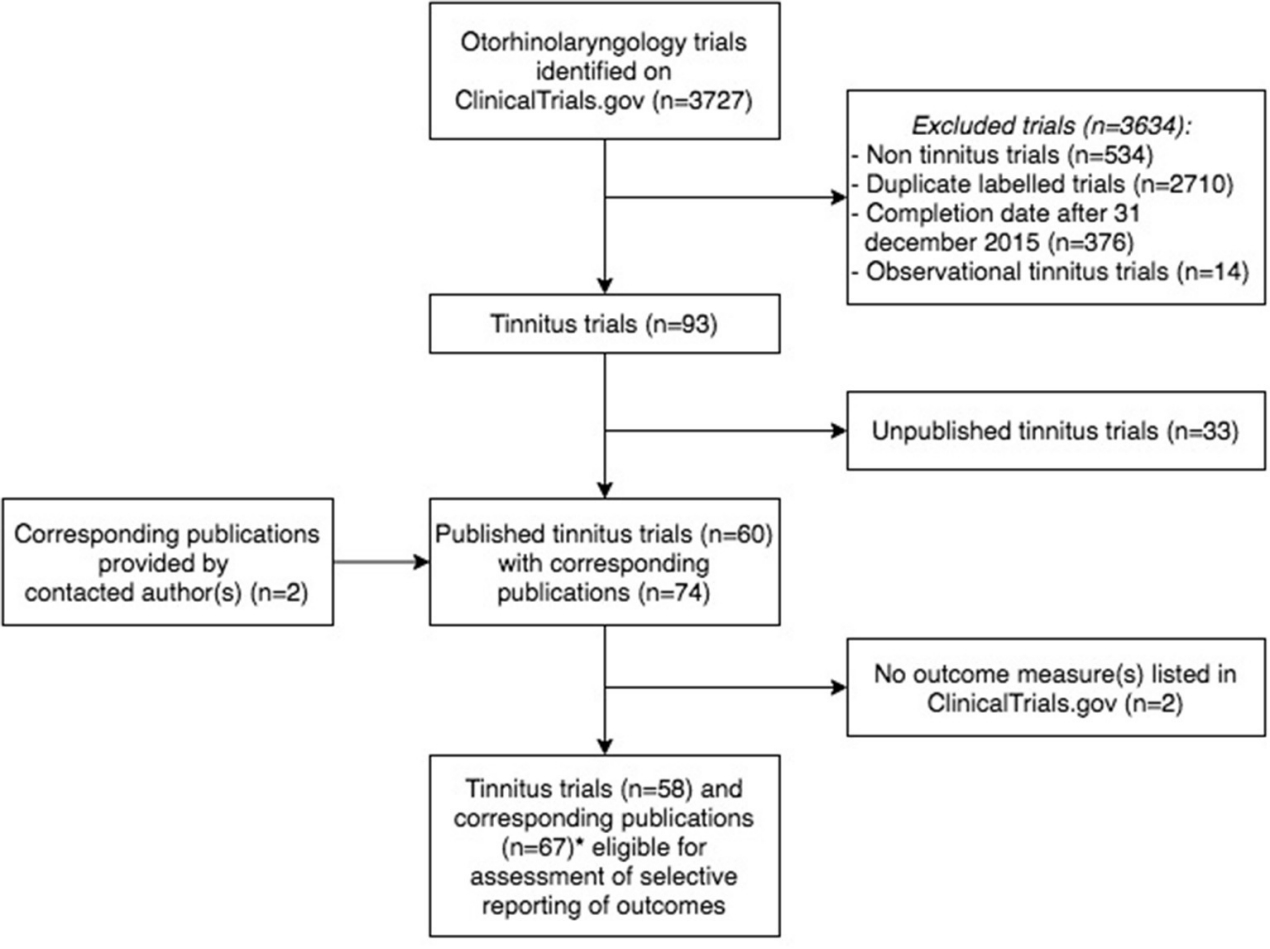
Flowchart of tinnitus trials and corresponding publications eligible for the assessment of outcome reporting bias. *Publications (*n* = 8) considered as one publication (*n* = 3), as these publications reported different outcome measures of the same trial (*n* = 3).

A total of 3.727 otorhinolaryngology trials were retrieved, of which 3.634 were excluded because they did not meet the inclusion criteria (Figure 1). In total, 93 tinnitus trials were included, with start dates from October 1999 to July 2015, which gave authors at least 2.5 years to publish trial results (Supplementary Dataset 1). The majority of trials were completed but did not present results on ClinicalTrials.gov. A total of 42 (45.2%) trials were registered prospectively, all started in or after July 2005, which is after the ICMJE stipulated that investigators must register their trial before patient enrollment. Trial characteristics are shown in Table 1. None of the trials included children. Of all tinnitus trials, 60 (64.5%) trials were published with 74 corresponding publications. In 43 (58.1%) of these publications, the NCT number of the accompanying trial was reported. The median time to publication was 23.0 months (IQR 14.0–41.0).

**Table 1:** Characteristics of included tinnitus trials.

		***n* (%) of trials (*N* = 93)**
Publication status	Published	60 (64.5)
	Unpublished	33 (35.5)
Recruitment status	Completed, has results	11 (11.8)
	Completed, no results available	63 (67.7)
	Terminated^a^	6 (6.5)
	Unkown^b^	9 (9.7)
	Recruiting	0 (0.0)
	Enrolling by invitation	1 (1.1)
	Active, not recruiting	0 (0.0)
	Withdrawn^c^	1 (1.1)
	Not yet recruiting	0 (0.0)
	Suspended^d^	2 (2.2)
Study topic	Psychology	13 (13.9)
	Sound therapy	8 (8.6)
	Pharmacology	30 (32.2)
	TMS	14 (15.1)
	Other	18 (19.4)
	Combined^e^	10 (10.8)
Funding	Industry	21 (22.6)
	Non-industry	69 (74.2)
Type of allocation	Randomized	72 (77.4)
	Non-randomized	8 (8.6)
	Missing	13 (14.0)
Trial registration	Prospective	42 (45.2)
	During patient enrolment	25 (26.9)
	Retrospective	19 (20.4)
	Missing	7 (7.5)
Year of trial registration^f^	Before July, 2005	8 (8.6)
	In and after July, 2005	83 (89.2)
	Missing	2 (2.2)
Trials with outcome measures	Primary	91 (97.9)
	Secondary	70 (75.3)
	None	2 (2.2)
Patient enrolment	Median (IQR)	54.0 (87.0)
	Missing	1 (1.1)
Trial duration	Median (IQR)	22.0 (21.0)
	Missing	11 (11.8)

*N, total sample size; n, number, IQR, Inter Quartile Range, TMS, transcranial magnetic stimulation*.

a *The study has stopped early and will not start again. Participants are no longer being examined or treated*.

b *A study on ClinicalTrials.gov whose last known status was recruiting; not yet recruiting; or active, not recruiting but that has passed its completion date, and the status has not been last verified within the past 2 years*.

c *The study stopped early, before enrolling its first participant*.

d *The study has stopped early but may start again*.

e *A combination of different topics is studied*.

f *In July 2005, the ICMJE stipulated that all investigators must register clinical trials in a qualifying public database before patient enrollment as a condition for publication*.

### Adherence to Study Record

Two of the 60 (3.3%) published trials did not prespecify any outcome measures and were therefore excluded for the assessment of selective reporting of outcome measures. Five publications reported different prespecified outcomes of the same trial and were therefore considered as one publication. This resulted in a comparison of 58 trials and 67 corresponding publications (Figure 1 and Supplementary Dataset 1). In total, 16 of the 67 (23.9%) included publications showed no discrepancy between the prespecified and published primary outcomes, and five (7.5%) publications reported outcomes entirely the same as both primary and secondary outcomes prespecified on CT.gov (Table 2). One included publication enclosed a reason for discrepancy in registered and reported outcomes. The authors mentioned that a different questionnaire was used because of a higher validity and greater responsiveness than the questionnaire they originally planned to use. Therefore, this discrepancy was not considered as such. Among the five non-discrepant publications, primary and secondary outcome measures were changed in CT.gov during patient enrolment (*n* = 1) and after trial publication (*n* = 1).

**Table 2:** Discrepancies between prespecified outcomes and reported outcomes in corresponding publications.

	***n* (%)**	***n* (%) of publications (*N* = 67)**
**Primary outcome**		
No discrepancy	35/85 (41.2)	16 (23.9)
Prespecified outcome not reported	11/85 (12.9)	10 (14.9)
Prespecified outcome “undefined”	21/85 (24.7)	19 (28.4)
Prespecified outcome downgraded to secondary outcome	4/85 (4.7)	4 (6.0)
Alteration in timing of assessment	27/77 (35.1)	20 (29.9)
Newly published outcome, not prespecified	8/77 (10.4)	5 (7.5)
**Secondary outcome**		
No discrepancy	77/198 (38.9)	5 (7.5)
Prespecified outcome not reported	57/198 (28.8)	24 (35.8)
Prespecified outcome “undefined”	38/198 (19.2)	23 (34.3)
Prespecified outcome upgraded to primary outcome	12/198 (6.1)	6 (9.0)
Alteration in timing of assessment	26/150 (17.3)	14 (20.9)
Newly published outcome, not prespecified	47/150 (31.3)	21 (31.3)
Newly published “undefined” outcome, not prespecified	38/316 (12.0)	17 (25.4)
Outcome not fully reported in ClinicalTrials.gov	19/283 (6.7)	–
No timing of assessment of outcome in Clinicaltrials.gov	24/283 (8.5)	–

*Note that outcome measures can have more than one discrepancy (e.g., prespecified outcome is undefined in the publication and has an altered timing of assessment). Note that publications can have more than one discrepancy*.

*N, total sample size; n, number; “undefined,” not reported as primary, nor as secondary outcome in the publication*.

**Figure 2:**
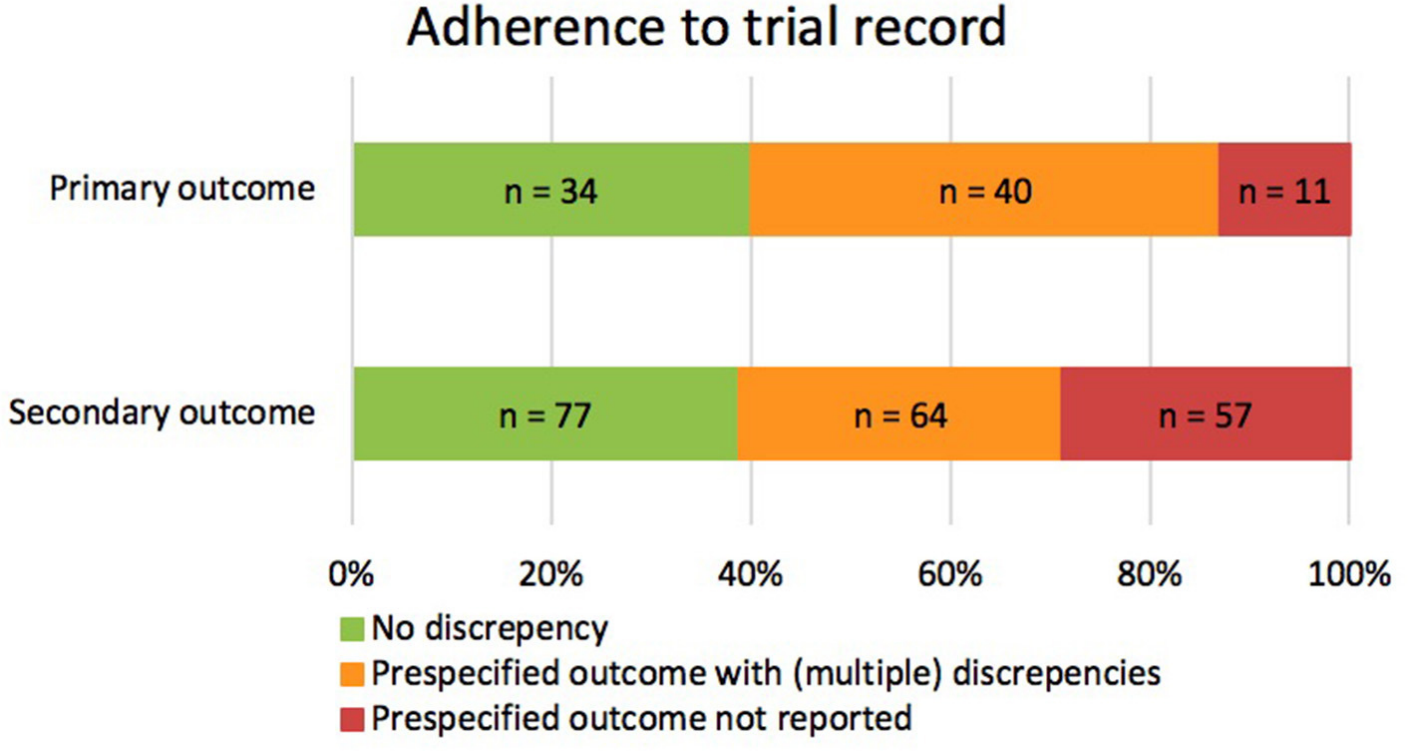
Consistencies and discrepancies between prespecified outcomes and reported outcomes in corresponding publications. The figure illustrates the correspondence between the prespecified and published primary and secondary outcomes. It includes prespecified outcomes that were published without discrepancy (green), with (multiple) discrepancies (orange), or that were not published at all (red).

Of the 85 prespecified primary outcomes (average of 1.27 per trial), 35 (41.2%) published their results as they were registered in clinicaltrials.gov, 39 (45.9%) were published with (multiple) discrepancies, and 11 (12.9%) were not published at all (Figure 2). The types of discrepancies are shown in Table 2. Of the 85 prespecified primary outcomes, four (4.7%) were downgraded to secondary outcomes in the publication, and in 27 (35.1%) of the prespecified primary outcomes, timing of assessment was changed.

Of the 198 prespecified secondary outcomes (average of 1.92 per trial), 77 (39.0%) were published as registered, 64 (32.3%) were published with (multiple) discrepancies, and 57 (28.8%) were not published at all (Figure 2). Among the prespecified secondary outcomes, 12 (6.1%) were upgraded to primary outcome measures in the publications (Table 2). Furthermore, 47 (31.3%) secondary outcomes were newly introduced, not being prespecified in CT.gov.

In 34 (50.7%) publications, authors failed to report outcome measures as primary or as secondary outcome measures (i.e., “undefined”). Of the prespecified outcome measures, 21 (24.7%) primary outcomes and 38 (19.2%) secondary outcomes remained undefined in the publications. A total of 38 undefined outcome measures were newly introduced and were not prespecified in ClinicalTrials.gov (Table 2).

### Discrepancies in Outcome Reporting According to Statistical Significance of Outcome Results

There was no statistically significant difference between the rate of non-discrepant and (any of our) discrepant primary outcomes, and for the statistical significance of its results (both in within- and between-group comparisons), there are no statistically significant differences (Table 3).

**Table 3:** Discrepancies between prespecified outcomes and reported outcomes in relation to statistical significance of its results.

	***n*** **(%) of discrepancies**									
	**Within-group comparison**					**Between-group comparison**				
	**Significant results**	**Non-significant results**	**Significance unclear**	***p*-value^**a**^**	**NA^**b**^/missing^**c**^**	**Significant results**	**Non- significant results**	**Significance unclear**	***p*-value^**a**^**	**NA^**b**^/missing^**c**^**
**Primary outcome**										
No discrepancy	16 (51.6)	9 (29.0)	6 (19.4)	–	4/0	13 (40.6)	16 (50.0)	3 (9.4)	–	3/0
Prespecified outcome not reported	–	–	–	–	–	–	–	–	–	–
Prespecified outcome “undefined”	11 (57.9)	3 (15.8)	5 (26.3)	0.55	0/2	5 (35.7)	5 (35.7)	4 (28.6)	0.24	6/1
Prespecified outcome downgraded to secondary outcome	3 (75.0)	1 (25.0)	0 (0.0)	0.56	0/0	1 (33.3)	1 (33.3)	1 (33.3)	0.46	1/0
Alteration in timing of assessment	16 (61.5)	3 (11.5)	7 (26.9)	0.27	0/1	11 (47.8)	7 (30.4)	5 (21.7)	0.25	3/1
Newly published outcome, not prespecified	5 (62.5)	0 (0.0)	3 (37.5)	0.19	0/0	0 (0.0)	2 (50.0)	2 (50.0)	0.05	4/0
**Secondary outcome**										
No discrepancy	32 (43.2)	32 (43.2)	10 (13.5)	–	1/2	24 (32.4)	45 (60.8)	5 (6.8)	–	2/1
Prespecified outcome not reported	–	–	–	–	–	–	–	–	–	–
Prespecified outcome “undefined”	16 (45.7)	11 (31.4)	8 (22.9)	0.35	1/2	12 (35.3)	9 (26.5)	13 (38.2)	<0.01^*^	2/2
Prespecified outcome upgraded to primary outcome	3 (27.3)	0 (0.0)	8 (72.7)	<0.01^*^	1/0	7 (63.6)	3 (27.3)	1 (9.1)	0.10	1/0
Alteration in timing of assessment	8 (33.3)	6 (25.0)	10 (41.7)	0.01^*^	2/0	12 (46.2)	10 (38.5)	4 (15.4)	0.11	0/0
Newly published outcome, not prespecified	10 (24.4)	14 (34.1)	17 (41.5)	0.03^*^	4/2	5 (16.7)	10 (33.3)	15 (50.0)	<0.01^*^	16/1
Newly published “undefined” outcome, not prespecified	12 (36.4)	10 (30.3)	11 (33.3)	0.07	4/1	9 (27.3)	17 (51.5)	7 (21.2)	0.08	4/1

*Note that outcome measures can have more than one discrepancy (e.g., prespecified outcome is undefined in the publication and has an altered timing of assessment). Note that publications can have more than one discrepancy. “Undefined,” not reported as primary, nor as secondary outcome in the publication; n, number; NA, not applicable;*

* *p < 0.05*.

a *Comparing the discrepancy rate of “significant,” “non-significant,” and “unclear” results of non-discrepant and discrepant (primary and/or secondary) outcome measures with a Chi-square test*.

b *No within- or between-group comparison was made due to trial design and type of outcome measure (e.g., number of adverse events)*.

c *No results were reported in the publication*.

The secondary outcomes did show statistical differences between the rate of non-discrepant and discrepant outcomes in relation to the statistical significance of its results (Table 3). In the within-group comparison, the results of the prespecified secondary outcomes that were upgraded to primary in the publication remained more often unclear [*n* = 8 (72.7%)] than the non-discrepant secondary outcomes [*n* = 10 (13.5%)] (*p* < 0.01). Similarly, the significance of the results of newly published secondary outcomes (that were not prespecified before) remained more often unclear [within-group comparison; *n* = 17 (41.5%) and between-group comparison; *n* = 15 (50.0%)] compared with non-discrepant secondary outcomes [within-group comparison; *n* = 10 (13.5%) and between-group comparison; *n* = 5 (6.8%)] (within- and between-group comparison; *p* < 0.01). There was a statistically significant difference in the rate of discrepancy in industry-funded [*n* = 98 (87.5%) discrepant outcomes] and non-industry funded trials [*n* = 172 (74.5%) discrepant outcomes] (*p* = 0.01).

There is a statistically significant positive correlation between the number of prespecified primary outcomes and the number of non-reported primary outcomes in the corresponding publication (Pearson's *r* = 0.62; *p* < 0.01). For secondary outcome measures, there is a statistically non-significant positive correlation (Pearson's *r* = 0.18; *p* = 0.13).

### Justification of Discrepancies From Authors

The authors of the publications with discrepancies were contacted by e-mail of which 19.4% (12/62) of the author's e-mail addresses were not in use anymore. Overall 22.6% (14/62) of the authors responded to the email, four of whom did not provide a justification for the discrepancy, and two of whom denied the existence of such discrepancies. Of the answers of the remaining eight authors, the reasons for discrepancies between the trial registry and publications varied from inattentiveness (*n* = 2), inexperience with trial registration (*n* = 2), and unawareness of the purpose of trial registration (*n* = 1). Other authors mentioned to have neglected to report all prespecified outcome measures on CT.gov (*n* = 1) or neglected to report change in outcome measures in the publication (*n* = 1). Other reasons were that authors wanted to focus exclusively on relevant facts (*n* = 2) or only use validated questionnaires and, therefore, neglected to report every prespecified outcome measure in the publication (*n* = 2). Finally, one author mentioned that the trial did not take place as planned, and therefore, outcome measures had to be altered.

### Changes From Initial to Final Registry

In 43 (74.1%) of the published trials, no changes were made in the registration of primary and secondary outcome measures in ClinicalTrials.gov. However, of these 43 trials, in 11 (25.6%) trials, the outcome measures were registered prospectively, 18 (41.7%) during patient enrollment and 13 (30.2%) retrospectively. The trialist of one trial did not report a study start date nor study completion date.

Among the 15 (25.9%) trialists that modified the registered outcome measures, eight (53.3%) trialists made changes during patient enrollment, three (20.0%) after trial completion, three (20.0%) after publication, and one (6.7%) trialist made changes during patient enrollment and after trial completion. Changes that were made before study initiation were not taken into consideration. Several amendments were made, with the majority being change in timing of assessment of primary [*n* = 9 (23.7%)] and secondary [*n* = 12 (31.6%)] outcome measures. A total of five (12.3%) primary and five (12.3%) secondary outcome measures were added after trial initiation (Table 4). None of the authors mentioned that prespecified outcomes had been revised after trial initiation.

**Table 4:** Updating of trial registry over time.

	***n*** **of amendments in trial registry**			
	**During patient enrollment (*N* = 23)**	**After trial completion (*N* = 8)**	**After publication (*N* = 6)**	**Total (%) (*N* = 38)**
**Primary outcome**				
Addition	2	1	2	5 (13.2)
Deletion	1	0	0	1 (2.6)
Downgrading to secondary outcome	2	1	1	4 (10.5)
Change in timing of assessment	5	2	2	9 (23.7)
**Secondary outcome**				
Addition	2	2	1	5 (13.2)
Deletion	0	0	0	0 (0.0)
Upgrading to primary outcome	1	0	0	1 (2.6)
Change in timing of assessment	10	2	0	12 (31.6)

## Discussion

We studied selective reporting in tinnitus trials by assessing discrepancies between outcomes prespecified in CT.gov and reported in corresponding publications. A total of 92.5% of the registered interventional tinnitus trials showed discrepancies in outcomes. Of all the prespecified primary and secondary outcomes, roughly two-thirds was published with multiple discrepancies or were not published at all. Both the statistical significance of results and funding source influenced the degree of selective outcome reporting. Approximately half of the trials were registered after trial initiation and frequent amendments were made in CT.gov in prespecified primary and secondary outcomes during or after patient enrollment.

In 76.1% of the publications, the primary outcome was discrepant compared with the prespecified outcome measure. This is a higher discrepancy rate than reported in previous studies comparing prespecified and published outcomes in other fields of medicine (5.9–46.6%) (8, 11–17, 21). The higher rate of discrepancy in the present study could be partly explained by the used methodology; we rated alteration of timing of assessment and outcome measures that remained “undefined” as discrepancies. Not all authors of similar studies included these as a discrepancy. Second, the discrepancies could reflect rather an inexperience with the submission process on CT.gov than to be caused by selective outcome reporting bias, since a relatively high number of trials started before or around 2005 (21). The rate of discrepancy of secondary outcomes in publications in our study (92.5%) is more in line with previous articles (44.0–90.0%) (8, 11–17, 21). Although registration quality was not the focus of this study, 6.7% of the outcomes where not fully reported in CT.gov, and 8.5% of the outcomes were lacking the timing of assessment. In light of the responses of the contacted authors, it seems that this could be due to inexperience with or unawareness of the purpose of trial registration. The lack of understanding of the importance to fully report prespecified outcome measures is supported by several studies that investigated selective outcome reporting by interviewing trialists (22–25).

Although our analysis does not entirely inquire all possible ethical considerations in the process from trial initiation to publication, publishing primary outcome measures discrepantly does not seem to be related to statistical significance of its results. However, for the secondary outcome measures, the statistical significance of the results influences the selective reporting. Similarly, our study showed that industry funding influences the likelihood of selective outcome reporting. Finally, unreported outcomes were common, with 12.9% of the prespecified primary and 28.8% of the prespecified secondary outcomes being omitted in the corresponding publications. Since the outcomes were omitted, it was not possible to relate it to the statistical significance of its results. However, an earlier study showed that one of the most commonly reported reasons for omitting outcomes was the lack of statistical significance (23). These findings are worrisome and imply that researchers make conscious choices on inclusion and exclusion of outcome measures during the trail or in the process of manuscript writing. This is partly emphasized by our analysis on the justification of discrepancies from authors. However, this was based on very limited data since only 22.6% of the authors responded to the queries. This “cherry-picking” might lead to biased and unimplementable results and distorts evidence, which clinicians have to use for clinical decision making (26). The rate of retrospective trial registration and the high number of amendments made in the trial register after trial initiation could be an additional source of selective outcome reporting bias. Unfortunately, none of the authors acknowledged these amendments in the corresponding publications. (Peer-)reviewers should be aware of this problem and should check the trial protocol and registry to ensure that authors have not altered the outcomes without appropriate justification (27).

This knowledge about selective reporting is important because it has impact on clinical care. Clinical trials are essential in providing information in clinical decision making, not only for individual clinicians but also for guideline making. Selective reporting and publication bias lead to an overestimation of beneficial effects and suppression of harmful effect of treatment (7, 8, 28). In treatment decision making, a clinician weighs these benefits and harm of a treatment. Selective reporting and publication bias distorts the balance between benefits and harms and, therefore, leads to wrong treatment decisions. Second, selective reporting is one of the problems that prevents us from finding an evidence-based treatment for multiple diseases, not only tinnitus. Altering or withholding results from the scientific community leads to research groups conducting similar trials and investigating the same outcomes all over the world. This not only leads to a waste of resources like funding and time but also to an increase in burden for included participants in those studies. The result of this study and papers like “increasing value, reducing waste” (5), and “why most published research findings are false” (29) leads to an increasing awareness on the importance of a transition in biomedical research to a more open and transparent scientific conduct. Selective reporting could be diminished if researchers are willing to be more transparent in their research protocol, methodology, and results.

A strength of our study is that we included a detailed evaluation of both primary and secondary outcomes. Second, we provided a minimum of 27 months for authors to publish trial data, which exceeds the median time of publication of otology trials (19). In addition, we included and combined all corresponding publications per trial in order to give a more realistic view of the reported outcomes, since some researchers may have chosen to publish outcomes in separate papers. Finally, we refined the understanding of selective outcome reporting by addressing the authors for justification of discrepancies and by analyzing amendments made in the trial registry over time. However, some limitations need to be acknowledged. Combining the multiple publications may have led to an overestimate of the reporting of primary outcome measures and an underestimate of the reporting of secondary outcome measures, a possibility that could not be everted on the basis of the current method. To minimize the risk of bias, all data extractions were done by two researchers independently. Still, it is possible that we have missed corresponding publications or have misinterpreted outcome measures in included papers.

In conclusion, incomplete reporting of outcomes within published articles of tinnitus trials is common and is associated with statistical non-significance and industry funding. Although the ICMJE registration policy increased the visibility of clinical trials, there is a further need of improvement in reporting of outcome measures and subsequently reducing research waste and improving research quality. Awareness of the presence of selective outcome reporting bias must be raised to limit the obstacles of finding an effective intervention for tinnitus.

## Data Availability Statement

The raw data supporting the conclusions of this article will be made available by the authors, without undue reservation.

## Author Contributions

IS, AS, IB, and JH contributed to conception and design of the study, and wrote sections of the manuscript. MB and IB organized the database. IS, IB, and JH performed the statistical analysis. IB and JH wrote the first draft of the manuscript. All authors contributed to manuscript revision, read, and approved the submitted version.

## Conflict of Interest

The authors declare that the research was conducted in the absence of any commercial or financial relationships that could be construed as a potential conflict of interest.
